# Early maternal separation impacts cognitive flexibility at the age of first independence in mice

**DOI:** 10.1016/j.dcn.2015.09.005

**Published:** 2015-10-19

**Authors:** A. Wren Thomas, Natalia Caporale, Claudia Wu, Linda Wilbrecht

**Affiliations:** a Helen Wills Neuroscience Graduate Program; b University of California Berkeley, Psychology Department; c University of California Irvine, Neurobiology and Behavior Graduate Program; d Helen Wills Neuroscience Institute

**Keywords:** Development, Reversal, Prefrontal, Perseveration, Stress, Neglect

## Abstract

Early life adversity is associated with increased risk for mental and physical health problems, including substance abuse. Changes in neural development caused by early life insults could cause or complicate these conditions. Maternal separation (MS) is a model of early adversity for rodents. Clear effects of MS have been shown on behavioral flexibility in rats, but studies of effects of MS on cognition in mice have been mixed. We hypothesized that previous studies focused on adult mice may have overlooked a developmental transition point when juvenile mice exhibit greater flexibility in reversal learning. Here, using a 4-choice reversal learning task we find that early MS leads to decreased flexibility in post-weaning juvenile mice, but no significant effects in adults. In a further study of voluntary ethanol consumption, we found that adult mice that had experienced MS showed greater cumulative 20% ethanol consumption in an intermittent access paradigm compared to controls. Our data confirm that the MS paradigm can reduce cognitive flexibility in mice and may enhance risk for substance abuse. We discuss possible interpretations of these data as stress-related impairment or adaptive earlier maturation in response to an adverse environment.

## Highlights

• MS mice tested in 4-choice task as juveniles are less flexible than littermates.
• MS mice tested in adulthood in 4-choice paradigm do not differ from littermates.
• MS mice showed greater ethanol consumption compared to littermates in adulthood.

## 1 Introduction

Early life experiences are known to have a profound impact on brain development and behavior. Epidemiological data and clinical studies suggest a strong link between childhood maltreatment and the development of substance use disorders, mental health disorders, obesity, and other physical health problems (Heim and Nemeroff, 2001, Sánchez et al., 2001, Fishbein et al., 2009, Felitti et al., 1998, Dube et al., 2001, Jaffee et al., 2002, Gilbert et al., 2009). Changes in neural circuits supporting executive function caused by early neglect or maltreatment could both cause and/or exacerbate mental and physical health conditions. For example, executive function deficits may contribute to the development and management of substance use disorders (Goldstein and Volkow, 2011).

Our goal here was to develop a mouse model of effects of early life adversity on executive function with a focus on the subdomain of cognitive flexibility (also called updating in the RDoc system). Additionally, we sought to investigate how early life stress might contribute to the development of addiction-related behaviors by assessing ethanol consumption using an intermittent access “drinking in the dark” paradigm that leads to binge drinking episodes in mice (Rhodes et al., 2005, Thiele and Navarro, 2014). We choose to focus on mice to enable use of the wealth of tools for the study of neural circuits that are currently most developed in this species.

There is a large body of work that has used rats as a model system to study the effects of early adverse experience on anxiety and fear behavior and also, to a lesser extent, cognitive function. Many of these models involve disruptions of the infant-mother relationship, which is thought to be one of the most important relationships in early life (Levine, 1962, Bowlby, 1982). Some studies have focused on comparing the offspring of mothers that provide low versus high levels of maternal care (Liu et al., 1997), while others have manipulated the amount of bedding to induce maternal stress and erratic behavior (Gilles et al., 1996; Ivy et al., 2008) or employed more invasive separation paradigms which remove pups from their mother during the early postnatal period (Plotsky and Meaney, 1993, Francis et al., 2002). The most invasive separation studies have used artificial rearing with no dam care at all (Lovic and Fleming, 2004). Rat pups that have experienced low care levels or maternal separation (MS) have been shown by a wealth of studies to exhibit altered stress reactivity and anxiety behavior (Liu et al., 1997, Huot et al., 2002, Ladd et al., 2004, Lippmann et al., 2007, Aisa et al., 2007, Pryce and Feldon, 2003, Gilles et al., 1996, Dalle Molle et al., 2012).

A small but growing body of work has found evidence of cognitive changes in rats following early adverse experience. Adult rats that were artificially-reared (with no dam contact) have been shown to exhibit impairments in tests of cognitive flexibility in a 2-choice attentional set shifting paradigm (ASST) in which rodents learn to dig for cereal reward in scented or textured material and the rewarded contingency is reversed or the rewarded dimension is shifted (Lovic and Fleming, 2004). Impairments in 2-choice reversal in this same digging based task have also been found in adult rats that underwent 3 h of maternal separation during the first two weeks of life (Baudin et al., 2012). Working memory and flexibility in spatial tasks has also been found to be altered in adolescent and adult rats following early maternal separation (Brenhouse and Andersen, 2011; but see Wang et al., 2015).

Notably, there is less evidence of a maternal separation effect on cognitive flexibility in mice, a species in which we have greater access to the study of specific circuits. Furthermore, past studies in mice have found inconsistent results which call into question the reliability of the rodent model. One study found the effects of maternal separation on cognitive function was strain-dependent (Mehta and Schmauss, 2011). The Balb/c strain showed spatial working memory and set-shifting deficits in adulthood following maternal separation, while the adult C57Bl/6 strain showed no impairments across multiple cognitive domains. Importantly, this mouse study found no deficit in reversal learning in either strain. In contrast, other studies have found adult spatial learning impairments and working memory impairments, including deficits in spatial reversal in both C57Bl/6J (Fabricius et al., 2008) and Balb/cJ strain (Wang et al., 2011), and Y maze spontaneous alternation and temporal order memory in the C57Bl6 strain (Yang et al., 2015). Two of these positive findings however focus on spatial tasks dependent on the hippocampus rather than odor or texture based digging tasks that have been found to be dependent on the integrity of the frontal cortex of rodents (Birrell and Brown, 2000, Garner et al., 2006, Bissonette et al., 2008, Kim and Ragozzino, 2005, McAlonan and Brown, 2003).

We have previously reported developmental changes in reversal learning in mice (Johnson and Wilbrecht, 2011) using a 4-choice, odor-based digging task (similar to the ASST but with 4 choices). We also established this task is dependent on the integrity of the dorsomedial frontal cortex (dmPFC) (Johnson and Wilbrecht, 2011). Designing this current study, we hypothesized that maternal separation may alter the developmental trajectory of cognitive flexibility, and that the 4-choice task may be more sensitive to detecting this effect in mice. Four-choice tasks likely produce greater cognitive load and ambiguity or uncertainty than 2-choice tasks and can be argued to be more naturalistic in their resemblance to real world foraging environments (Ragozzino and Rozman, 2007). We chose to use the C57Bl/6 strain of mice for this study, as it is commonly used in labs that study neural circuits and behavior.

Exposure to early life stressful events has been postulated to be associated with increased vulnerability to develop substance use disorders. Human subject studies have found an association between early life adversity and increased incidence of alcohol use disorders (Macmillan et al., 2001, Green et al., 2010, Enoch, 2011). Maternal separation in rats (Huot et al., 2001) and mice (Cruz et al., 2008) has been shown to lead to increased ethanol consumption in adulthood, but the ethanol consumption paradigms used have differed and one study suggests handling protocols may impact later drinking (Ploj et al., 2003, Moffett et al., 2007). We sought to confirm if maternal separation experience in mice leads to greater consumption of ethanol using a more recent “drinking in the dark” model (Rhodes et al., 2005) that promotes binge-levels of drinking.

Here, we report that a fairly low dose of 60 min per day of maternal separation in mice for the first ten days of life can impact reversal performance measured using a 4-choice odor based task in juvenile mice tested at postnatal day 26. However, when testing in adulthood (∼P60), early maternal separation experience did not lead to any difference in performance in the 4-choice reversal task, even after the daily separation time was raised to 180 min. We discuss how a change in the developmental trajectory of cognitive function may be interpreted as adaptive earlier maturation in adverse circumstances or stress induced impairment. Our data also strengthen links between early adversity and substance use disorders. When adult MS and littermate control mice were allowed intermittent access to 20% ethanol, we found a significant interaction between MS experience x time on cumulative ethanol consumption.

## 2 Materials and methods

### 2.1 Animals

Male and female C57Bl/6 *Mus musculus* (lines originally obtained from Charles River) were used for this study. Dams and sires were housed in pairs throughout the breeding and rearing period. Post-weaning, experimental mice were group housed in single sex cages, 2–5 per cage. All cages were on a 12/12 reverse light cycle (lights off at 10 AM). Testing took place during the dark period. All animals received nesting material and plastic hut in their home cage. All procedures were approved by the Ernest Gallo Clinic and Research Center and UC Berkeley Animal Care and Use Committees.

#### 2.1.1 Maternal separation (MS)

From postnatal (P) day 1 to P11, with a one day break on the weekend for some litters, half of each litter of pups was removed daily from their cage for either 60 or 180 min (MS group), while half of the litter remained with the dam (littermate, LM group). (Group sizes: Cohort 1 Juvenile 60 min: MS = 14, LM = 15; Cohort 2 Adult 60 min: MS = 12, LM = 8; Cohort 3 Adult 180 min: MS = 19, LM = 11). This particular paradigm was selected as a combination of elements from a broad variety of MS protocols used in other rodent studies (Bock et al., 2005, Monroy et al., 2010, Macri et al., 2008). During separation, MS pups were kept in a clean cage warmed by an electric pad. MS pups were able to hear and smell each other, but not touch during seperation due to a cardboard divider. The control group were littermates (LM group), forming the other half of the litter who were initially handled and marked the same as the MS half, but who were placed back in the home cage with the dam during the period of separation. All mice were identified by marker and then ear clipping. After P11 mice were not handled until weaning at P21, with the exception of one weekly cage cleaning. At weaning, the average weight of maternally separated mice was not distinguishable from littermates (MS = 8.99 ± 0.34, *n* = 14, LM = 8.83 ± 0.35 *n* = 15; *t* (27) = 0.3258, *P* = 0.75, cohort 1).

### 2.2 Four-choice odor discrimination and reversal task

**Fig. 1:**
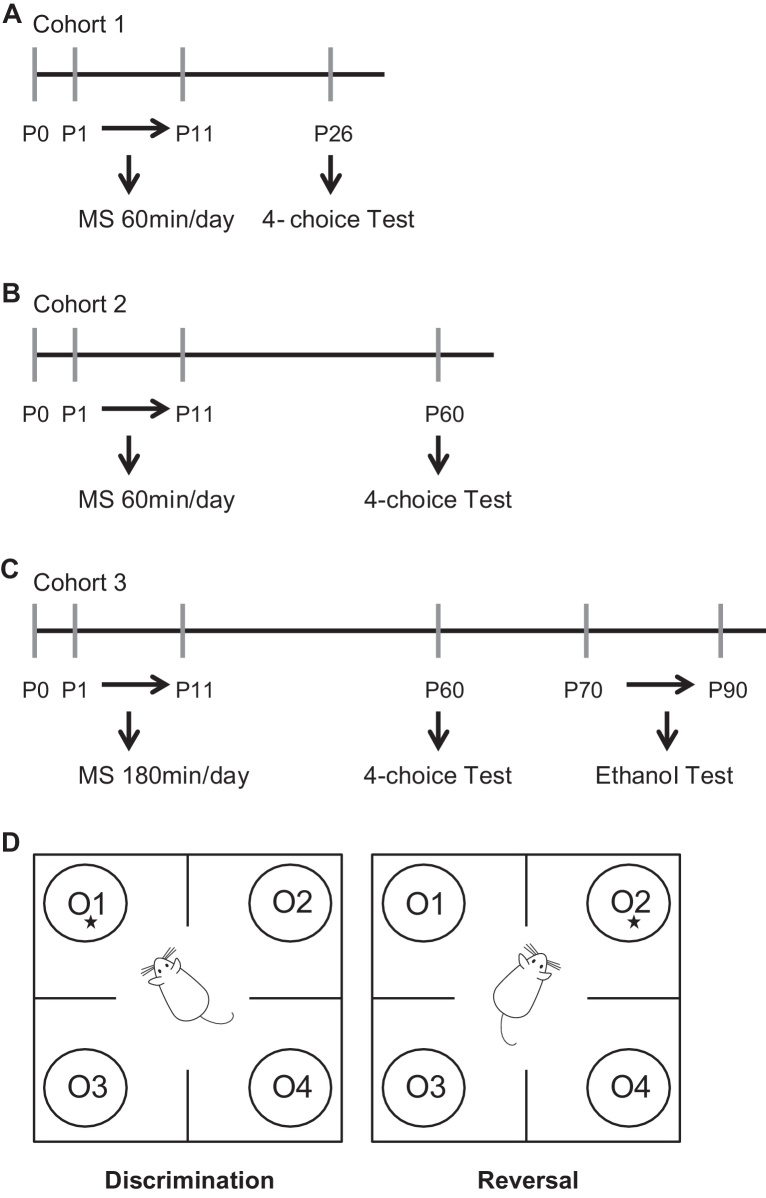
Experimental timeline and set-up. (A) Timeline of maternal separation paradigm and 4-choice testing for juvenile 60 min (B) adult 60 min maternal separation and (C) adult 180 min maternal separation and ethanol drinking paradigm. (D) Schematic of arena in 4-choice task. Mice learned to discriminate among four odors to learn which odor contained a buried cheerio reward. In the reversal phase, a previously incorrect odor predicted the reward location. All odor locations were shuffled each trial.

Extensive methods for the four-choice reversal were published previously (Johnson and Wilbrecht, 2011). Briefly, testing took place over 5 days with 2 days of food deprivation, followed by habituation, shaping and one final day of discrimination and reversal testing. Juvenile mice started food deprivation at P22 and were tested at P26 (Fig. 1a.), while adult mice started food deprivation at P56 and were tested at P60 (Fig. 1b and c.). The same cohorts of mice that underwent the maternal separation or littermate procedure were food deprived (to 90% of *ad libitum* of adult weight or 90% of peer weights if testing before P50). On habituation day, mice were placed in the behavioral testing box and consumed a novel cereal reward (Cheerio fragments, General Mills) for 30 min. On shaping day, mice learned to retrieve cereal pieces from the bottom of a single bowl that was increasingly filled with unscented shavings. On the main testing day, mice were presented with 4 bowls (all sham baited with a screen over a cheerio), each containing shavings of different scents (odors 1-4: anise, clove, litsea, thyme, respectively). Only one bowl contained an accessible reward (Fig. 1d). This was Odor 1 during the discrimination phase and Odor 2 during the reversal phase. During each trial mice were allowed 3 min to explore the arena and indicate a single choice by purposively digging in a bowl with both paws. Bowl locations were changed each trial, so mice were forced to use odor to reliably retrieve the cereal reward. An absence of a choice during the 3 min was scored as an omission, and mice were returned to a start cylinder and a new trial was initiated. Upon reaching criterion of 8/10 sequential trials correct during the discrimination phase, the reversal phase began on the next trial in the same session. During this phase, Odor 2 was now rewarded and a novel odor replaced Odor 4 (eucalyptus). Perseverative errors were scored as choices to dig in the bowl with the previously rewarded odor (Odor 1). Irrelevant errors were choices to dig in the bowl with odor 3, which was present during both phases of the behavior. Novel errors were choices to dig in the newly introduced 4th odor, which were not rewarded.

### 2.3 Intermittent-access ethanol consumption (single bottle paradigm) procedure

**Fig. 5:**
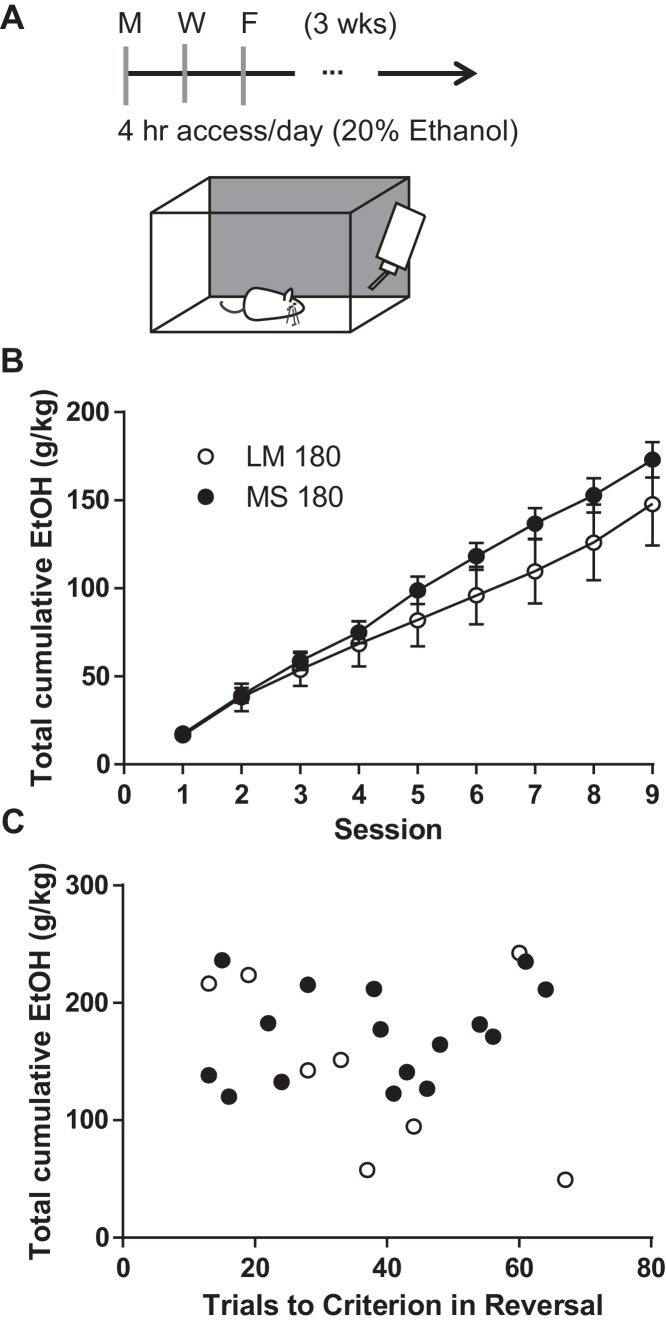
Maternal separation (MS180) enhanced cumulative alcohol consumption compared to littermate controls. (A) Schematic of ethanol paradigm (B) Cumulative ethanol consumption across 9 sessions. A two-way repeated measures ANOVA revealed a significant interaction of MS180 experience and session (*P* < 0.05) (C) No correlation was found between 4-choice reversal performance (trials to criterion) and cumulative ethanol consumption on Day 9 (*r* = −0.032, *P* = .88). Bars represent mean ± SEM.

The intermittent access “drinking in the dark” ethanol consumption model was chosen since it produces binge drinking behavior in which rodents will often attain blood alcohol levels comparable to the legal limits for driving after ethanol consumption in humans (Rhodes et al., 2005, Thiele and Navarro, 2014). To measure ethanol consumption, mice that had undergone 180 minutes of maternal separation or littermate experience and then were tested on the 4-choice reversal task were moved to single housing at ∼P70 (Fig. 1c). (MS = 16; LM = 9; The initiation of the ethanol experiment was delayed and so 3 MS mice and 2 LM mice from cohort 3 were not tested on ethanol consumption). Mice had ad lib access to water except during testing when the water bottle was replaced with 20% ethanol solution during a 4 hour test session conducted from 1 PM to 5 PM in the reverse dark light cycle (which started at 10 AM). Ad lib water was available until 1 PM and then after 5 PM. Testing was complete after three weeks with a total of 9 sessions conducted on Monday, Wednesday and Friday (Fig. 5a). Bottles and mice were weighed after each session to calculate ethanol consumption (ethanol per kg mouse weight). Blood was sampled from a tail vein after the 9th session for a subset of the mice (*n* = 4–6 per group) to measure blood alcohol content (BAC).

### 2.4 Statistical analysis

Values are reported as mean (*M*) ± SEM. Data were tested for normal distribution and two-tailed *t* tests or ANOVAS, followed by post-hoc analysis were used for all statistical comparisons. Statistical significance was set at *P* < 0.05; analysis and graphing were performed with GraphPad Prism v6.

## 3 Results

### 3.1 Four-choice odor discrimination and reversal: Juveniles (60 Minute Separation)-Cohort 1

**Fig. 2:**
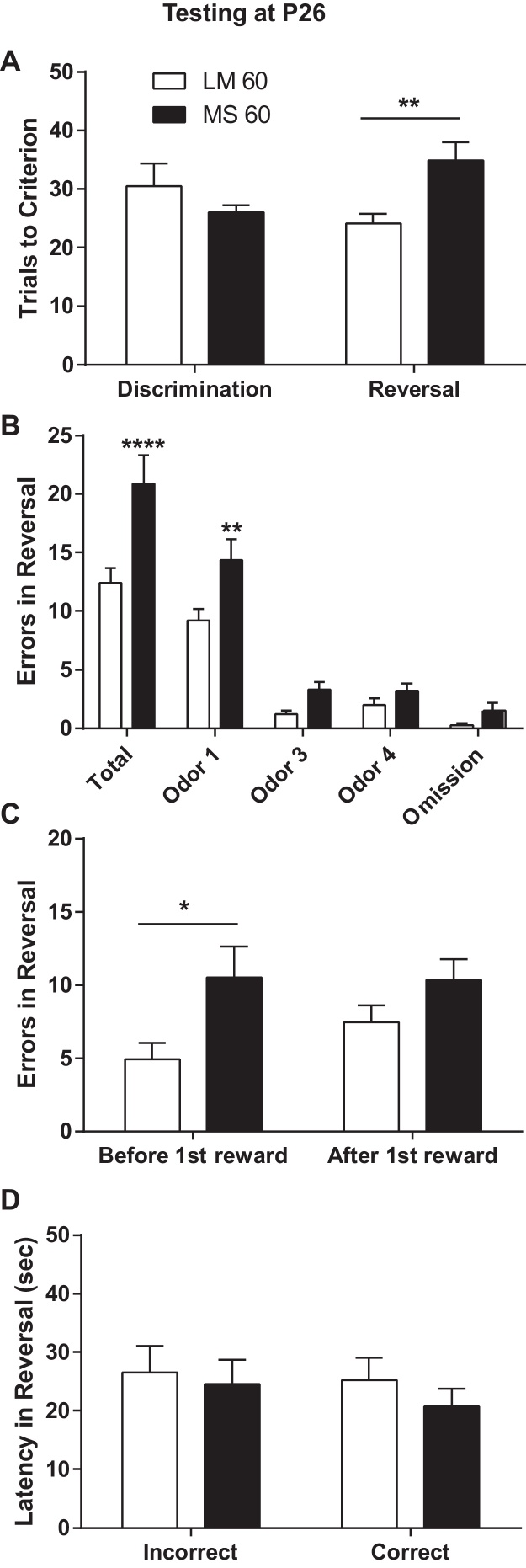
Maternally separated (MS60) mice tested at a juvenile stage show less behavioral flexibility in a 4-choice odor discrimination and reversal task compared to littermate (LM60) controls. (A) Juvenile MS60 (*N* = 14) and LM60 mice (*N* = 15) did not differ in the total trials to reach criterion in the discrimination phase (*P* = 0.30). In the reversal phase, MS60 mice required significantly more trials to reach criterion compared to littermates (*P* < 0.01). (B) Analysis of error type revealed that MS60 mice made more total errors (*P* < 0.0001) and odor 1 errors (*P* < 0.01) compared to littermates. (C) MS60 mice made more errors prior to the first reward than littermates (*P* < 0.05). (D) MS60 mice did not differ from littermates in the latency to dig in incorrect (*P* = 0.75) or correct trials (*P* = 0.37). Bars represent mean ± SEM *P < 0.05; **P < 0.01, ***P < 0.001, ****P < 0.0001.

To explore how maternal separation influences behavioral flexibility during development we compared performance of mice that had undergone 60 min of maternal separation (MS60) to littermate controls (LM60) on a 4-choice odor discrimination and reversal task tested at a juvenile age (P25-P26). During the discrimination phase, animals learned to dig for a buried cheerio reward in bowls with differently scented shavings. Only one bowl was rewarded. Maternally separated (*N* = 14) mice did not differ from littermates (*N* = 15) in the discrimination phase of the task [littermate: *M* = 30.47 ± 3.95; maternal separation: *M* = 26.00 ± 1.27; *t*(27) = 1.05, *P* = 0.30] (Fig. 2a). However, in the reversal phase, in which a previously incorrect odor predicted the location of the reward, maternally separated mice required significantly more trials to reach criterion compared to littermates [*t*(27) = 3.02, *P* < 0.01] (Fig. 2a). Analysis of error type using a two-way ANOVA revealed a significant main effect of MS experience [*F*(1,135) = 25.38, *P* < 0.0001] and error-type [*F*(4,135) = 75.04, *P* < 0.0001] and a significant interaction of maternal experience and error type [*F*(4,135) = 3.82, *P* = 0.0057] (Fig. 2b). Bonferroni post-hoc tests found that the MS mice made more total errors (*P* < 0.0001) and odor 1 errors (*P* < 0.01) compared to littermates (Fig. 2b). Analyzing errors made before or after the first reward revealed that MS mice made significantly more errors before the first reward compared to LM mice [*t*(27) = 2.38, *P* < 0.05], but there was no difference in the number of errors made after the first reward [*t*(27) = 1.62, *P* = 0.12] (Fig. 2c). Maternally separated mice did not differ from littermates in latency to dig in incorrect [*t*(27) = 0.32, *P* = 0.75) or correct trials [*t*(27) = 0.92, *P* = 0.37) (Fig. 2d).

### 3.2 Four-choice odor discrimination and reversal: Adults (60 Minute Separation)-Cohort 2

**Fig. 3:**
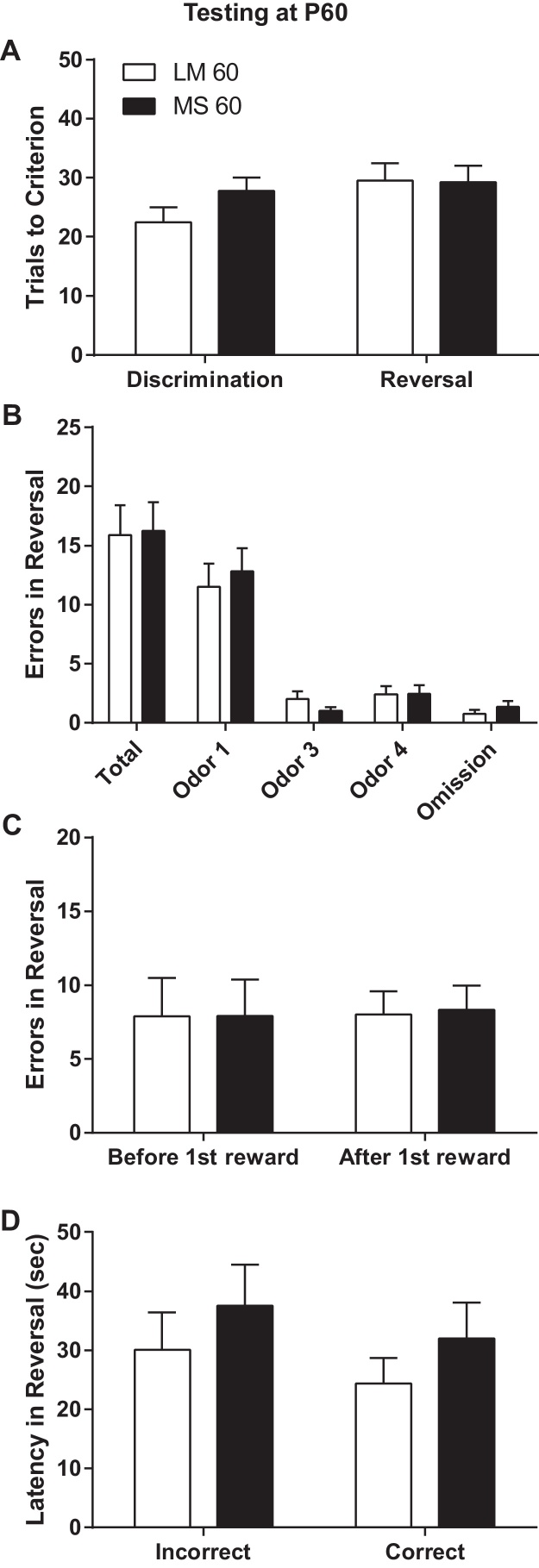
Maternally separated mice (MS 60) tested in adulthood do not differ from littermates (LM 60) in the 4-choice task (A) Maternally separated (*N* = 12) adult mice did not differ from littermates (*N* = 8) in the total trials to criterion in the discrimination phase (*P* = 0.15), or reversal phase (*P* = 0.95). (B) Analysis of error type using a two-way ANOVA revealed a main effect of error type (*P* < 0.0001) but no main effect of maternal experience (*P* = 0.78) or interaction (*P* = 0.96). (C) There was no difference between MS and LM mice in the number of errors made before the first reward (*P* = 0.99) or after first reward (*P* = 0.89). (D) Latency to dig did not differ between groups in incorrect trials (*P* = 0.46) or correct trials (*P* = 0.36). Bars represent mean ± SEM.

To test the impact of maternal separation on adult reversal performance, a second cohort of mice underwent the same 60 min maternal separation (*N* = 12) and littermate (*N* = 8) procedure from P1-11 as above, and then were tested on the 4-choice task in adulthood (P59-P60). Adult maternal separation mice did not differ from littermates in the total trials to criterion in the discrimination phase [*t*(18) = 1.49, *P* = 0.15) (Fig. 3a). In contrast to maternally separated mice tested as juveniles, there was no difference between adult maternally separated mice and littermates in the number of trials it took to reach criterion in the reversal phase [*t*(18) = 0.06, *P* = 0.95) (Fig. 3a). Analysis of error type using a two-way ANOVA revealed a main effect of error type [*F*(4, 90) = 41.32, *P* < 0.0001], but no main effect of MS experience [*F*(1, 90) = 0.08, *P* = 0.78] or interaction [*F*(4, 90) = 0.15, *P* = 0.96] (Fig. 3b). There was no difference between MS and LM mice in the number of errors made before the first reward [*t*(18) = 0.01, *P* = 0.99] or after first reward [*t*(18) = 0.14, *P* = 0.89] (Fig. 3c). Additionally, latency to dig did not differ between groups in incorrect trials [*t*(18) = 0.76, *P* = 0.46] or correct trials [*t*(18) = 0.93, *P* = 0.36] (Fig. 3d).

### 3.3 Four-choice odor discrimination and reversal: Adults (180 Minute Separation)-Cohort 3

**Fig. 4:**
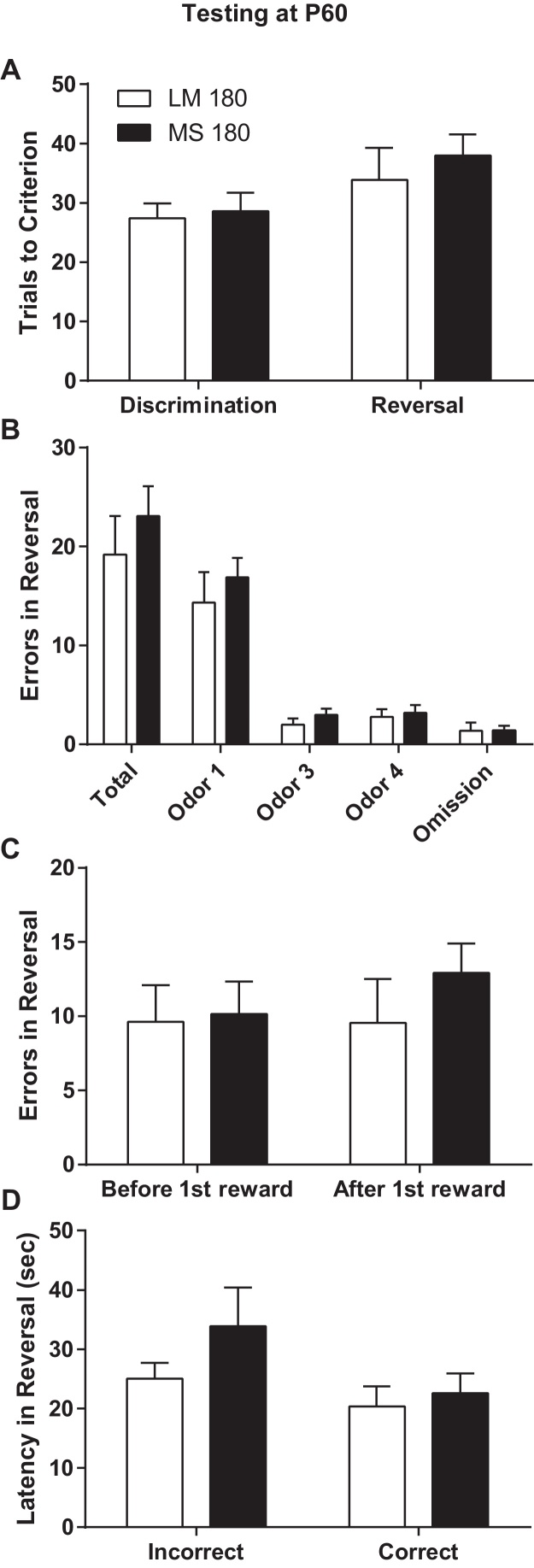
A longer daily separation dose does not result in differences between maternally separated (*N* = 19) mice and littermates (*N* = 11) tested on the 4-choice task in adulthood (A) Adult MS180 did not differ from littermates in the discrimination phase (*P* = 0.78) and reversal phase of the 4-choice task (*P* = 0.51). (B) Analysis of error type using a two-way ANOVA revealed a main effect of error type (*P* < 0.0001) but no main effect of maternal experience (*P* = 0.21) or interaction (*P* = 0.86). (C) There was no difference between MS180 and LM180 groups in the number errors made prior to the first reward (*P* = 0.88) or after first reward (*P* = 0.33). (D) There was no difference in latency to dig between MS180 and LM180 mice in incorrect (*P* = 0.33) and correct trials (*P* = 0.66). Bars represent mean ± SEM.

We next ran a third cohort of animals in the maternal separation (*N* = 19) and littermate (*N* = 11) paradigm, increasing the separation period to 180 min a day from P1-11 (MS180). Again, mice were tested using the 4-choice task in adulthood (P59-P60). Similar to the adults that had undergone maternal separation for 60 min, we found no difference in the total trials to criterion in the discrimination phase [*t*(28) = 0.28, *P* = 0.78], and in the reversal phase [*t*(28) = 0.66, *P* = 0.51] between the 180 min maternal separation group and littermates tested as adults (Fig. 4a). Analysis of error type using a two-way ANOVA revealed a main effect of error type [*F*(4, 140) = 40.75, *P* < 0.0001], but no main effect of MS180 experience [*F*(1, 140) = 1.58, *P* = 0.21] or interaction [*F*(4, 140) = 0.32, *P* = 0.86] (Fig. 4b). There was no difference between MS180 and LM groups in the number of errors made prior to the first reward [*t*(28) = 0.15, *P* = 0.88] or after first reward [*t*(28) = 1.00, *P* = 0.33] (Fig. 4c). There was no difference in latency to dig between maternally separated and littermate adult mice in incorrect [*t*(28) = 0.99, *P* = 0.33] and correct trials [*t*(28) = 0.45, *P* = 0.66] (Fig. 4d).

### 3.4 Intermittent-access 20% ethanol consumption: Adults (180 Minute Separation)- Cohort 3

The same cohort of animals that underwent maternal separation or littermate experience for 180 min and were tested on the 4-choice task in adulthood (P59-P60), were next tested on ethanol consumption using an intermittent-access ethanol drinking paradigm starting at ∼P70 (MS = 16; LM = 9) (Fig. 5a). A two-way repeated measures ANOVA revealed a main effect of session [*F*(8, 184) = 156.3, *P* < 0.0001] and a significant interaction of MS180 experience and session [*F*(8, 184) = 2.17; *P* < 0.05] with MS180 treated mice drinking more than LM180 controls over time (Fig. 5b). No correlation was found between reversal performance and cumulative ethanol intake [r = −0.032, *P* = .88] (Fig. 5c). Blood ethanol concentration taken at the end of the final 3 h drinking period revealed mice with ranges from 0.004–0.149% with a median of 0.039% and group average of .045 ± .027%, for the LM group (*n* = 4) and .056 ± 0.026% for the MS group (*n* = 6). For reference, the legal driving limit in humans is set at 0.08% in the United States. As mice may have reached higher levels earlier in the 3 hour period when blood was not sampled, these blood values should be interpreted with caution.

## 4 Discussion

Our goal was to use a maternal separation paradigm to determine how an early life insult impacts the development of cognitive flexibility in mice. We found that maternally separated mice tested at a juvenile age (P25-P26) showed less flexibility in learning to reverse a previously learned association compared to littermates. Maternally separated mice required more trials to reach criterion (Fig. 2a) and made more errors than littermates, specifically during the reversal phase of the task (Fig. 2b). These data indicate that maternal separation induces changes in cognitive flexibility, an important domain of executive function early in life when mice have just been weaned and must forage for their own food. Notably, we found that juvenile MS mice made more errors prior to the first reward compared to littermates (Fig. 2c). This increase in perseveration compared to littermates could result from insensitivity to disappointing outcomes leading to lack of updating or difficulty inhibiting their responses to the previously rewarded odor. However, once the first reward in the new odor was experienced, maternally separated and littermate juvenile mice did not differ in the number of errors made, suggesting that MS mice and littermate controls updated behavior at a similar rate once positive feedback was encountered.

We did not detect any significant effects of MS on reversal performance in the 4-choice task when mice were tested in adulthood (Fig. 3), even if the daily separation period was extended from 60 min to 180 min (Fig. 4). This could suggest that early maternal separation has no lasting consequences on decision-making and underlying neural circuits in mice despite extensive evidence of changes in executive function related circuitry in rats (Pascual and Zamora-León, 2007, Chocyk et al., 2010, Monroy et al., 2010, Uchida et al., 2010, Muhammad and Kolb, 2011, Brenhouse et al., 2013, Anier et al., 2014) and degus (Helmeke et al., 2008). This species difference seems unlikely. Yang et al. (2015) recently reported that early maternal stress driven by inadequate supply of bedding produces lasting effects on neuronal morphology in the anterior cingulate cortex in P75 mice and this stress led to impairments in tests of working memory.

We also find that in adulthood, mice that had experienced early postnatal maternal separation had higher cumulative ethanol consumption compared to littermates in an intermittent access 20% ethanol single bottle choice “drinking in the dark” paradigm (Fig. 5), suggesting that at least some differences in reward and decision making circuits remain in mice into adulthood. Our ethanol data are consistent with a previous study that found that a 180 min maternal separation paradigm increases 10% ethanol intake in a daily three bottle choice paradigm and also increases 6% and 10% ethanol consumption in a daily operant paradigm in adult mice (Cruz et al., 2008). Our ethanol consumption data, acquired using a different paradigm which is able to promote binge-like levels of ethanol drinking in short periods of time, strengthen these previous findings, which together strongly suggest that early maternal separation enhances voluntary ethanol consumption in adulthood. This adds to growing evidence that early adversity may enhance risk for the development of substance use disorders.

Our results may also explain why previous studies found no effect of early MS on reversal learning in mice. If, as we observe, the MS effect on flexibility is limited to juvenile development in mice, then effects may have been missed by previous researchers that only tested adults. Task design may also be a factor. Prior studies of MS in mice that found no effect on reversal used the 2-choice attentional set shifting task (Mehta and Schmauss, 2011) while our current study used a 4-choice paradigm. Prior work in rats has shown that the 4-choice reversal task is more difficult to learn than the 2-choice task (Kim and Ragozzino, 2005, Ragozzino et al., 2003, Ragozzino and Rozman, 2007) placing greater cognitive load on the animal to sort out the best of the multiple options. Testing mice with this 4-choice task may have made MS effects easier to detect in mice even when using a supposedly more “stress resistant” (Mehta and Schmauss, 2011) C57Bl/6 strain.

Although rats and mice both show similar cognitive differences as a result of early MS, it is notable the age at which MS effects become apparent differs by species. Here we show effects in mice on cognitive flexibility are limited to the juvenile period, while in the literature rats have shown MS effects that emerge after the juvenile period (post P30). For example, working memory deficits assessed using the radial arm maze were observed in maternally separated rats tested at P40, but not at P25 (Brenhouse and Andersen, 2011). Differences in MS rats and controls also emerged after P30 in the Morris water maze (Wang et al., 2015). In this latter study, spatial reversal notably became more flexible in adolescent and adult MS rats compared to controls. This variability in MS effects on cognition suggests there are complex interrelationships between species, age and the specific cognitive domain tested by a task (e.g. spatial vs. non-spatial).

It may help to frame this variability in the context of two models that are used to discuss how the brain and body respond to stress (Hostinar and Gunnar, 2013). In one, the allostatic load model (McEwen and Stellar, 1993), repeated stress has negative impacts on the brain. In another, the adaptive calibration model (Del Giudice, Ellis and Shirtcliff, 2011), stress calibrates or tunes developmental processes to allow an animal to match its behavior to the environment. The allostatic load model attempts to explain how organisms respond to insults, positing that adaptations, in the short-term, can provide benefits, however if experienced for longer periods can lead to negative consequences (Hostinar and Gunnar, 2013). The adaptive calibration model attempts to answer why systems act the way they do in the context of life history strategies instead of viewing certain outcomes as dysfunctional or pathological.

Under the umbrella of adaptive calibration model, there is a growing body of literature suggesting that early social deprivation may accelerate the maturation of threat related behavior. A series of studies focused on extinction and recovery of fear conditioning memory have noted that rats exposed to MS show early adult-like fear extinction and recovery behavior (Callaghan and Richardson, 2011). Stress in the early homecage has also been shown to alter the developmental trajectory of attachment and avoidance learning (Moriceau et al., 2009). This has been echoed by discovery of adult-like functional connectivity in the amygdala-ventromedial PFC of children who were institutionalized during early life (Gee et al., 2013).

Consistent with the earlier maturation hypothesis, the juvenile MS mice tested in our 4-choice reversal paradigm performed in a manner indistinguishable from littermate adults (reversal trials to criterion: MS juvenile *M* = 34.86 ± 3.20, LM adults (LM 60) *M* = 29.50 ± 2.98, *t*(20) = 1.11, *P* = .3) or untreated adults from previously published data (Control adults *M* = 36.18 ± 4.78, *t*(23) = 0.24, *P* = .8; data from Johnson and Wilbrecht, 2011). The age at which we observe a significant effect of MS (P25-26) is likely a transitional phase of life in which the animal is moving from parental dependence to independence and in which the animal must quickly learn about its changing environment and make adjustments based on those experiences (Spear, 2000). In our prior study which described a developmental decrease in flexibility in a 4-choice reversal task (Johnson and Wilbrecht, 2011), we hypothesized that this heightened flexibility observed in juveniles may be particularly important for navigating the ambiguous and/or uncertain environment that an animal faces at this unique life stage. Periods of enhanced flexibility have been observed in other developmental studies in rats (Simon and Moghaddam, 2015) and humans (Van Der Schaaf et al., 2011). Interpreted in the context of the adaptive calibration model and the earlier maturation hypothesis, our current MS results could suggest that in the face of adversity it might be adaptive for mice to use more perseverative, adult-like strategies during the juvenile period.

Our results can also be interpreted in the context of the chronic stress and allostatic load model. We can compare our MS data to studies of chronic restraint stress, that have found repeated stress in adulthood can lead to alteration in frontal circuit neural morphology (Radley et al., 2004, Radley et al., 2009) and disrupt cognitive flexibility in the 2-choice ASST task (Liston et al., 2006). Although chronic restraint stress in adulthood selectively affected attentional set shifting (changing a rule from odor to texture) and not reversal learning (switching from odor 1 to odor 2) (Liston et al., 2006), it is possible that early MS might simply impair circuit function underlying reversal learning in an analogous fashion. Loss of function could thus be quite different than acceleration of maturation. In summary, both the allostatic load and adaptive calibration model could be used to explain our current data. We speculate that different strategies adopted at the time of dispersal (whether evolutionarily adaptive or maladaptive) could potentially influence the criteria and timing of territory and reproductive decisions that could influence the whole life trajectory of a wild living rodent. This idea could also be applied to human society and decision making in its greater complexity. Future work on the biological changes following MS in rodents should shed light on the appropriateness of the adaptive calibration model versus the allostatic load model and help guide translational efforts to ameliorate the impact of early life adversity on human health.

## Conflict of interest

The authors declare that there are no conflicts of interest.
